# Lower cognitive control network connectivity in stroke participants with depressive features

**DOI:** 10.1038/s41398-017-0038-x

**Published:** 2017-12-08

**Authors:** Natalia Egorova, Toby Cumming, Chris Shirbin, Michele Veldsman, Emilio Werden, Amy Brodtmann

**Affiliations:** The Florey Institute of Neuroscience and Mental Health, Melbourne, VIC Australia

**Keywords:** Learning and memory, Depression

## Abstract

Around one-third of people develop depression following ischaemic stroke, yet the underlying mechanisms are poorly understood. Post-stroke depression has been linked to frontal infarcts, mainly lesions in the left dorsolateral prefrontal cortex (DLPFC). But depression is a network disorder that cannot be fully characterised through lesion-symptom mapping. Researchers of depression in non-stroke populations have successfully tapped into the cognitive control network (CCN) using the bilateral DLPFC as a seed, and found that CCN resting-state connectivity is reduced in even mildly depressed subjects, compared to healthy controls. Hence, we aimed to investigate the association between post-stroke depressive features and the CCN resting-state connectivity in a stroke population. We analysed DLPFC resting-state connectivity in 64 stroke participants, 20 of whom showed depressive features assessed with the Patient Health Questionnaire (PHQ-9) at 3 months after stroke. We directly compared groups showing symptoms of depression with those who did not, and performed a regression with PHQ-9 scores in all participants, controlling for age, gender, lesion volume and stroke severity. Post-stroke depression was associated with lower connectivity between the left DLPFC and the right supramarginal gyrus (SMG) in both group and regression analyses. Neither the seed nor the results overlapped with stroke lesions. These findings confirm an important role of the left DLPFC in post-stroke depression, but now show that large-scale network disruptions following stroke associated with depressive features occur without lesions in the DLPFC.

## Introduction

Depression is a psychiatric disorder associated with persistent sadness, loss of energy and cognitive impairment^1,2^. It develops in about 30% of stroke patients, regardless of stroke site or severity^3^. Post-stroke depression has been shown to negatively impact on stroke recovery, worsening cognitive outcomes and reducing motivation^4^.

The neuroanatomical changes that underlie the clinical manifestation of post-stroke depression remain unclear^5^. In the acute post-stroke phase, frontal and temporal lesions have been related to depressed mood^6,7^. Among the frontal regions, lesions to the dorsolateral prefrontal cortex (DLPFC) have been frequently associated with depression^8,9^. Both left or right DLPFC lesions have been implicated, depending on the post-stroke stage studied (i.e., acute, subacute or chronic)^10^. However, a recent study by Grajny et al. directly correlated depression severity to the extent of damage in the left DLPFC^8^. Information about the prevalence of lesions is crucial for understanding post-stroke depression; however, lesion-based characterisation has major limitations. The explanatory power of stroke lesion size and location in accounting for the depressive phenotype is higher in the acute stroke phases, while at later stages lesion mapping might be less reliable due to brain plasticity during the recovery process^11,12^. In addition, while lesion location is a worse predictor for higher cognitive functions, such as attention and memory, network-based connectivity measures are better suited to describe impairment in more complex behaviours^13^. Many authors posit that complex psychiatric disorders such as depression would be better characterised using brain network measures that also account for functional and structural brain reorganisation in the subacute and chronic stroke phases.

Resting-state functional magnetic resonance imaging (rs-fMRI) has been shown to be valuable for characterising stroke and recovery^14^. rs-fMRI was utilised in several studies to identify differences between depressed and healthy subjects or to correlate brain changes to depression severity. They found that post-stroke depression is associated with decreased connectivity in the default mode network^15,16^ and increased connectivity in the affective network^17^. Cognitive control network (CCN) is a set of brain regions, localised primarily in the frontal and parietal cortices, that interact in a coupled manner to implement cognitive control in a variety of tasks^18^. Despite the evidence from lesion studies pointing to the significant role of the DLPFC in post-stroke depression, the CCN has not been investigated. Yet, studies in non-stroke depressed patients with cognitive deficits suggest that bilateral DLPFC connectivity plays an important role in both major^19^ and subthreshold depression^20^. We investigated resting-state connectivity in the CCN in participants with and without depressive features 3 months after stroke. In light of the suggested specificity of the left DLPFC in depressed stroke participants, we considered the left and right DLPFC connectivity separately.

## Materials and methods

### Participants

Participants with ischaemic stroke were recruited from the Stroke Units at three Melbourne hospitals: Austin Hospital, Box Hill Hospital and the Royal Melbourne Hospital as part of the Cognition and Neocortical Volume after Stroke (CANVAS) study^21^. Each hospital’s ethics committee approved the study in line with the Declaration of Helsinki. The details of the protocol are described in detail in ref. ^21^. Participants with psychiatric history prior to stroke were excluded from the study. This was a core exclusion criterion for the entire CANVAS study.

### Outcome measures

The severity of participants’ stroke was assessed with the National Institutes of Health Stroke Scale (NIHSS) examination performed at hospital admission. The severity of participants’ depression was assessed with the Patient Health Questionnaire-9^22^ specifically validated for the use in stroke populations^23^. The PHQ-9 scores each of the nine DSM-5 criteria as 0 (not at all) to 3 (nearly every day); scores of 0–27 are possible. The scores of 5–9, 10–14, 15–19 and ≥20 represent mild, moderate, moderately severe and severe depression, respectively (scores correlate with depression severity). DSM-5 defines post-stroke mood disorders as mood disorders due to stroke with depressive features, major depressive-like episode or mixed-mood features^24^. While the PHQ-9 is not a clinical diagnostic tool for depression, it allows identifying the presence of depressive features. Participants in this study did not have previous history of depression and were not medicated for depression at the time of the study.

### Imaging data acquisition and pre-processing

All images were acquired on a Siemens 3 T Tim Trio scanner (Erlangen, Germany) with a 32-channel head coil. As part of an ongoing longitudinal study, participants were assessed at 3 months after their stroke. A high-resolution anatomical MPRAGE was collected (volume of 160 sagittal slices with 1 mm isotropic voxels, repetition time (TR) = 1900 ms, echo time (TE) = 2.55 ms, 9° flip angle, 100% field of view in the phase direction and 256 × 256 acquisition matrix). A high-resolution 3D SPACE-FLAIR image was acquired (with 160 1-mm-thick sagittal slices, TR = 6000 ms, TE = 380 ms, 120° flip angle, 100% field of view in the phase direction and 256 × 254 acquisition matrix). Resting-state data were obtained (132 volumes taking ~7 min, with axial oriented, interleaved slices, 3 mm isotropic voxels, 3 mm slice gap, TR = 3000 ms, TE = 30 ms and 85° flip angle, 100% field of view in phase direction and 72 × 72 acquisition matrix). During resting-state acquisition participants were instructed to keep their eyes closed.

Functional images were pre-processed in SPM8 (Wellcome Department of Imaging Neuroscience, London, UK, http://www.fil.ion.ucl.ac.uk/spm/). The pre-processing pipeline included slice-time correction, with the middle slice as a reference, six-parameter rigid body realignment to estimate and correct for movement, and co-registration to the high-resolution structural image.

Lesions were manually traced on the high-resolution FLAIR image. A stroke neurologist (A.B.) visually inspected and verified the manually traced images. A binary lesion mask was created to improve patient image segmentation and normalisation to the MNI152 template using the Clinical Toolbox SPM extension^25^ to improve tissue segmentation and preserve the lesion size. Tissue segmentations were manually inspected for quality assurance. Functional images were smoothed with an 8-mm full width half maximum Gaussian kernel.

Pre-processed images were imported into the Conn Toolbox version 16b. Additional head motion analysis was performed using the artefact detection toolbox (ART; https://www.nitrc.org/projects/artifact_detect/). Time points were marked as outliers if global signal exceeded three SDs from the mean and if movement exceeded 0.5 mm of scan-to-scan deviation. ART regressors were added to the six-rigid body motion regressors for motion estimation. Lesion-masked segmentations were used for noise correction. Data were bandpass-filtered between 0.008 and 0.09 Hz. Nuisance regression and bandpass-filtering were performed simultaneously using the Simult function in the Conn Toolbox.

Total intracranial volumes (TIV) were estimated using FreeSurfer automatic segmentation (http://surfer.nmr.mgh.harvard.edu/). White matter hyperintensities (WMHs) were segmented using a Bayesian probabilities approach from the combined information of the T1 and FLAIR images^26^.

### Seeds for the rs-fMRI analysis

For the rs-fMRI analysis we used the seed applied in several resting-state connectivity studies for elucidating the CCN network in major depression^19^ and subthreshold depression^20^: the bilateral dorsolateral prefrontal cortex (DLPFC), MNI coordinates ±36, 27, 29, spheres with a 3-mm radius. However, unlike in the previous studies using one seed combining left and right DLPFC, we computed the left and right DLPFC connectivity networks separately. Both region of interest (ROIs) were drawn in WFU Pickatlas^27^.

### Lesion analysis

**Fig. 1:**
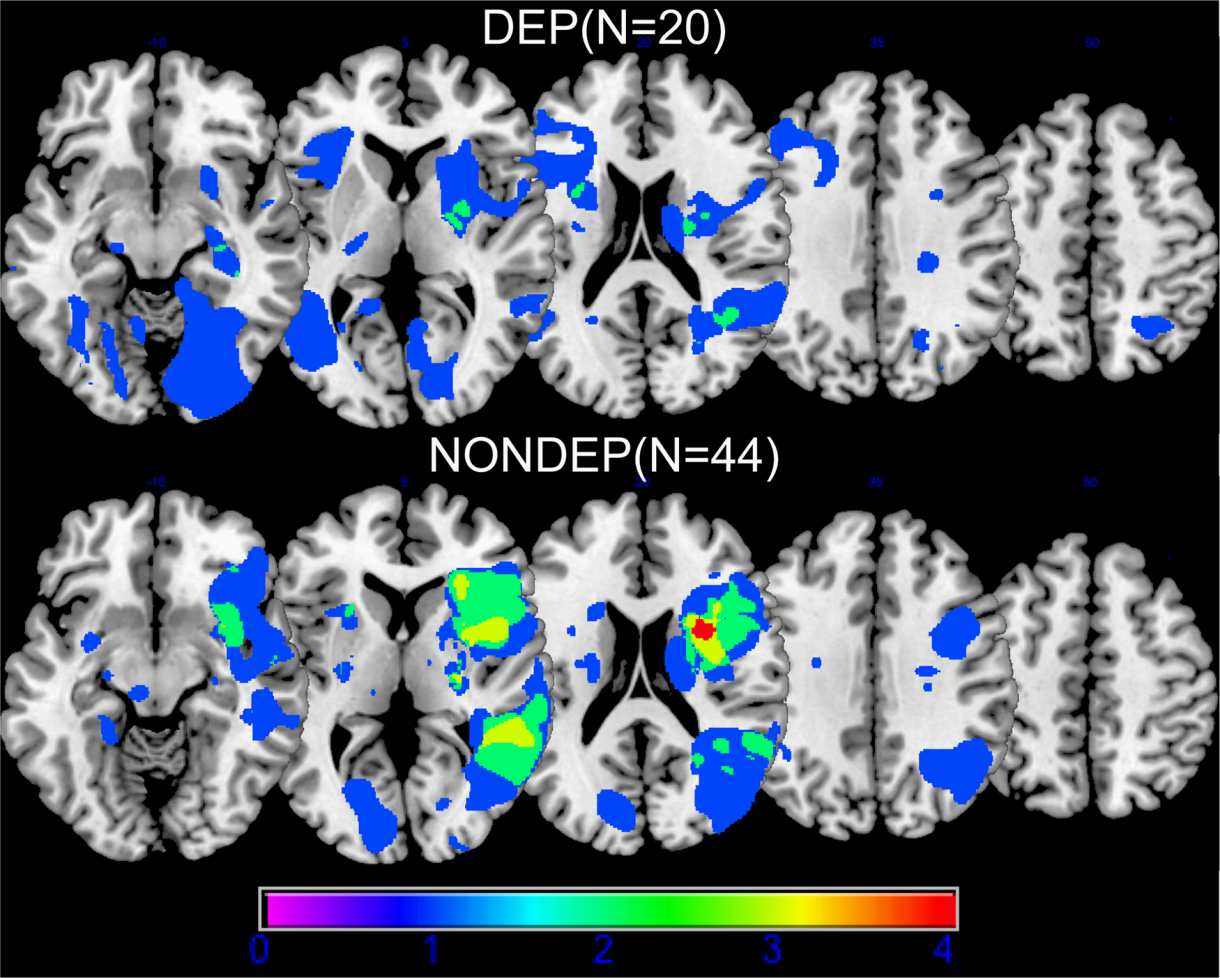
Lesion overlap Lesion overlap for DEP (scale 0–4 overlapping subjects) and NONDEP (scale 0–4 overlapping subjects) participants

Lesion overlap images were prepared using MRIcron software^28^. We created the overlap maps for 20 DEP and 44 NONDEP participants (Fig. 1).

### Statistical analysis

#### Behavioural data

We identified participants who had PHQ-9 score of ≥5 as a cutoff for mild depression at 3 months post stroke. We divided all stroke participants into two groups: those with depressive features (DEP; ≥5) and those without (NONDEP; <5), and compared the two groups on a number of behavioural variables, as well as brain measures including TIV and the extent of WMH, which have been associated with stroke and depression^29–31^. We used a two-sample *t*-test for age, PHQ-9 at 3 months, TIV, WMH volume, Boston naming test, Digit Span test, Hopkins Verbal Learning test, Star cancellation test, Complex figure test (copy), Complex figure test (recall); Wilcoxon Test for NIHSS at baseline and a *Χ*^2^-test for sex. Full details on these group comparisons are reported in Table 1.

**Table 1:** Group comparison

Variable	NONDEP	DEP	*p* value (two-tailed)
*Demographic variables*			
PHQ-9 at 3 months—M (SD)	2.07 (1.4)	8.4 (3.8)	0.00 (by design)
Number of subjects and sex—N (*N* female)	44 (11)	20 (12)	0.01
NIHSS at baseline—median (range)	2 (0–10)	3 (1–10)	0.12
Age—M (SD)	67.72 (14.5)	67.55 (10.7)	0.96
*Brain measures*			
Total intracranial volume (ml)—M (SD)	1511 (166)	1453 (149)	0.17
White matter hyperintensity volume (ml)—M (SD)	9.44 (11.21)	9.48 (14.17)	0.99
Lesion volume (ml)—M (SD)	6.08 (1.86)	8.38 (2.62)	0.48
*Cognitive assessment*			
Boston Naming Test *Z-*score—M (SD)	0.42 (0.83)	0.30 (0.74)	0.58
Digit Span Z-score—M (SD)	0.06 (0.81)	−0.35 (0.94)	0.11
Hopkins verbal learning test total *Z*-score—M (SD)	0.02 (1.21)	0.29 (1.01)	0.35
Star Cancellation Test score—M (SD)	53.3 (1.43)	53.4 (1.04)	0.76
The Rey–Osterrieth complex figure Test Copy Z score—M (SD)	0.05 (1.34)	−0.15 (1.29)	0.32
The Rey–Osterrieth complex figure Test Recall Z score—M (SD)	−0.40 (1.79)	−0.07 (1.00)	0.78

*P* values for the *t*-test (PHQ-9 at 3 months, age, total intracranial volume, white matter hyperintensity volume, Boston naming test, Digit Span test, Hopkins Verbal Learning test, Star cancellation test, Complex figure test (copy), Complex figure test (recall); for Wilcoxon test (NIHSS at baseline); for *Χ*^2^-test (sex).

*NONDEP* group without depressive features, *DEP* group with depressive features, *M* mean, *SD* standard deviation, *N* number

#### Imaging data

We directly compared the connectivity of the right and left DLPFC between stroke participants showing depressive features (DEP) and those who did not (NONDEP). We also performed a whole-brain regression analysis of DLPFC connectivity with PHQ-9 scores, controlling for age, gender and baseline NIHSS scores in all participants. The threshold of voxel-wise *p* < 0.001 corrected for multiple comparisons at the cluster level *p*_FDR_ < 0.05 was used for assessing statistical significance of results.

## Results

### Behavioural results

The first 64 participants in the CANVAS study who completed assessment at 3 months and had a full set of imaging data and depression scores available were included in the analysis. On average, subjects in this study had a stroke of minor severity, as is evident in the baseline NIHSS scores (median = 2, range 0–10). At 3 months, when depression was measured, median NIHSS score was 0 (range 0–5): i.e., no stroke symptoms. Twenty participants presented with mild-to-moderate depressive features (DEP, PHQ-9 score of above 5) at 3 months. This equated to an expected 30% prevalence rate. Forty-four participants had the score of less than 5 and were considered ‘non-depressed’ (NONDEP). The groups were not different on age or NIHSS baseline scores—see Table 1. Stroke participants in our sample presented with minor stroke, as assessed by the NIHSS. There were significantly more women in the DEP group compared to the NONDEP group (see Table 1). This is consistent with previous literature^32^. The groups were also not different in their performance on several cognitive tests, including the Boston naming test, Digit Span test, Hopkins Verbal Learning test, Star cancellation test, Complex figure test (copy), Complex figure test (recall), see Table 1.

### rs-fMRI group comparison at 3 months

**Fig. 2:**
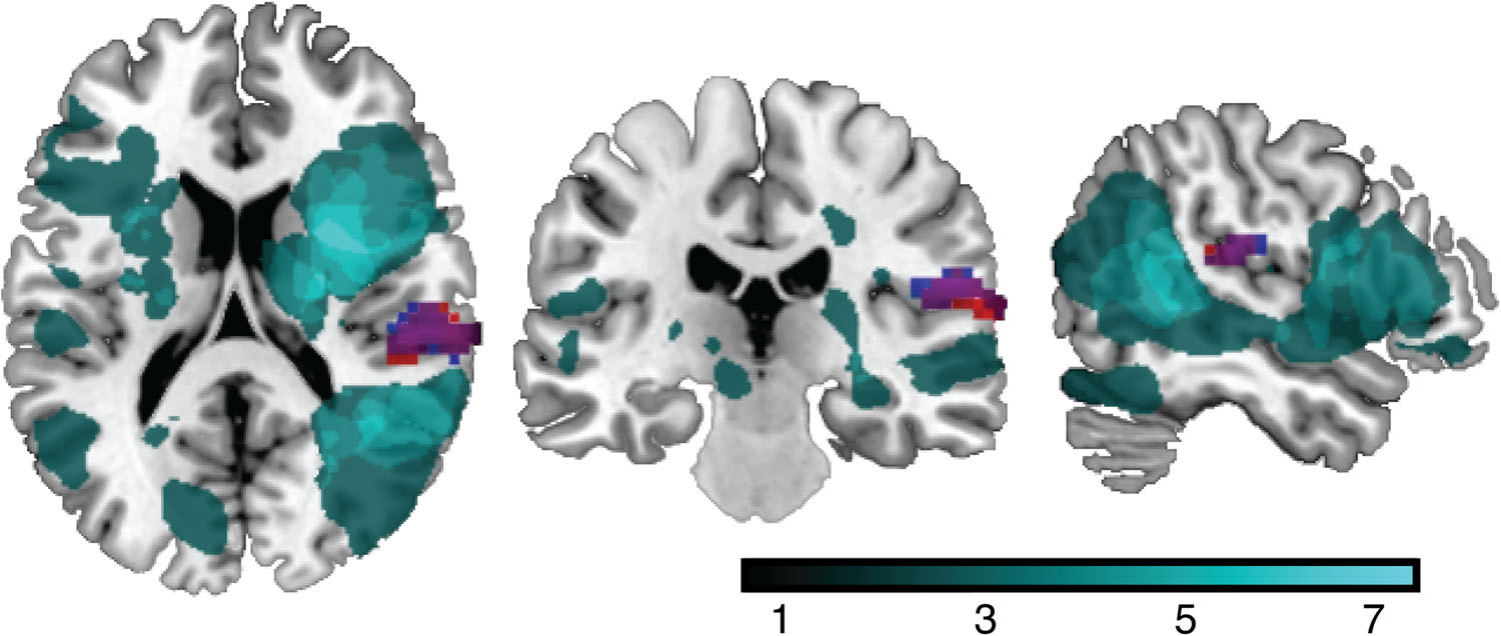
Main results Lesion overlap in all stroke participants *N* = 64 shown in cyan. A cluster in the right supramarginal gyrus (MNI: + 66, −24, 15; *p* = 0.001, cluster-corrected *p*_FDR_ = 0.007, *k* = 103) shows lower connectivity of the left DLPFC in a direct comparison between the DEP (*N* = 20) vs. NONDEP (*N* = 44) participants (shown in red); shown in blue is a negative association between the connectivity of the left DLPFC and right supramarginal gyrus with the PHQ-9 scores at 3 months in all subjects (MNI: 57, −21, 21, *p* < 0.001, cluster-corrected at *p*_FDR_ = 0.01, *k* = 110); shown in purple is an overlap between the group results and the regression results

Using the right DLPFC seed, no significant differences in connectivity were observed between groups. Using the left DLPFC seed, significantly lower connectivity with a cluster (*k* = 103) in the right supramarginal gyrus (SMG) was observed in the DEP group (cluster centre (MNI:+66, −24, 15) voxel-wise *p* = 0.001, cluster-corrected *p*_FDR_ = 0.007). Figure 2 shows the significant cluster in red. In addition, we found that the groups did not differ in TIV, as well as in the extent of WMHs.

### Assessing depression severity in all participants

We also performed a regression analysis with the PHQ-9 score for all 64 participants, regardless of depressive feature status, controlling for age, gender, and baseline NIHSS for the left and right DLPFC seeds separately. Higher PHQ-9 scores at 3 months were associated with lower connectivity between the left DLPFC and the right SMG (cluster centre (MNI: 57, −21, 21) significant at the voxel level *p* < 0.001, cluster-corrected at p_FDR_ = 0.01, *k* = 110). Figure 2 shows the cluster in blue; note the overlap between the group and correlation results in purple.

### Overlap with stroke lesions

No overlap between the stroke lesions and the right DLPFC seed was observed. One participant presented with a lesion in the left DLPFC overlapping with the seed. This particular participant had a PHQ-9 score of 6, placing them in the DEP (‘mild’ depression based on the PHQ-9 severity scale) group. Removing this participant’s data from all analyses did not change the results. The cluster in the right SMG observed in both correlation and group comparison results did not overlap with lesions in any subject (Fig. 2). Lesion volume did not significantly differ between groups (Table 1).

## Discussion

We investigated the CCN connectivity in participants with and without depressive features at 3 months post stroke and found that left DLPFC–right SMG connectivity was lower in DEP subjects compared to the NONDEP group. Importantly, the connectivity values negatively correlated with depressive feature severity in all participants at 3 months.

### Specifically left-lateralised DLPFC connectivity in post-stroke depression

On the one hand, evidence from prior lesion studies attested to the crucial role of the left DLFPC in post-stroke depression^8,33^, especially depression characterised by cognitive impairment^34^. On the other hand, findings from non-stroke populations demonstrated aberrant connectivity of the DLPFC and the CCN in depression^19,20,35,36^. The results of our study provide a bridge between these previous findings, demonstrating that even in the absence of lesions in the left DLPFC, stroke participants can experience depressive symptoms related to lower DLPFC connectivity with the SMG. These results are also consistent with our recent report of aberrant low-frequency fluctuations specifically in the left DLPFC in post-stroke depression^37^.

Lower connectivity at 3 months post stroke in the left, but not in the right, DLPFC represents additional evidence for using left DLPFC over right DLPFC as a target for clinical interventions, for instance, in non-invasive brain stimulation. In fact, in non-stroke participants, the left DLPFC has already been a preferred target for transcranial magnetic stimulation as a treatment of depression, although the efficacy was shown to depend on the exact location and potential functional connectivity with the subgenual anterior cingulate cortex^38^. The left DLPFC has also been a target for transcranial direct current stimulation, specifically aimed at ameliorating cognitive control in depression^39^. Based on the relevance of the left DLPFC connectivity to the severity of post-stroke depression in our study, we speculate that the left DLPFC might be a suitable target for neuromodulation in post-stroke depression.

### Sensitivity to mild post-stroke depression

Lower resting-state CCN connectivity found to correlate with depressive feature severity in this study could be a sensitive biomarker of even mild post-stroke depression. Most subjects in our DEP group endorsed features of only mild-to-moderate depression. Lower DLPFC–SMG connectivity using the same DLPFC seed has been previously reported in subjects with subthreshold depression compared to healthy controls, albeit using bilateral seeds^20^. In the study by Hwang et al., the depressed vs. non-depressed group comparison of DLPFC connectivity revealed a bilateral SMG cluster overlapping with the right SMG cluster reported here. The depression severity regression in their study, however, showed a left-lateralised SMG, unlike our right SMG result. Taken together, the results of both studies suggest that DLPFC connectivity to both left and right SMG could be important in mild depression, with the laterality differences likely explained by the use of the bilateral vs. left DLPFC seed.

Reported CCN connectivity differences have been observed, despite no significant differences in the level of cognitive performance between groups. Previous studies, such as those in Alzheimer’s disease, showed that brain changes often precede behavioural cognitive decline^40^. Given that changes in CCN connectivity were sensitive to relatively mild depressive symptoms, future studies could investigate feasibility of using CCN connectivity for predicting cognitive decline in depressed stroke participants.

### DLPFC–SMG connectivity

Cognitive dysfunction in depression has been described in terms of both cognitive biases, such as fixation on negative information^41^, as well as impaired general executive functions; e.g., deficits in cognitive control that prevent depressed patients from overcoming maladaptive biases. CCN is known to be critically involved in top–down modulation of attention and working memory and comprises the lateral prefrontal and inferior parietal cortices^42^. Little is known how the interaction between these regions emerges to produce higher cognitive function. It is likely shaped by both the underlying physical neuroanatomical connections between frontal and parietal areas (structural connectivity) and by correlated activity in distinct distant regions (functional connectivity). The DLPFC is an important node of this network, frequently used to derive the functional CCN in resting-state studies, and was therefore used as a seed here. The SMG is located at the border of the parietal and temporal cortices, and together with the angular gyrus forms the temporoparietal junction, which has been shown to be involved in memory, language, attention and social processing^43^. The DLPFC is known to have long-range structural connections with the parietal cortex, and the SMG in particular^44^. Equally, both DLPFC and SMG are highly connected functionally. For example, the right SMG and lower DLPFC–rSMG connectivity have been previously linked to egocentricity^45,46^ that can be related to self-focus observed in depression, and to depression in general^20,47^. The patterns of intrinsic connectivity of the temporoparietal junction (including to the DLPFC) reveal connections to salience, attention and social networks^48^. Therefore, the DLPFC–SMG connectivity could be particularly vulnerable to depression. Both DLPFC and temporoparietal junction represent so-called ‘rich-club’ nodes in the brain network organisation, and are postulated as being affected following stroke^49^.

### Limitations

The main limitation of our study is that correlation analyses between depression and functional connectivity cannot reveal the direction of influence of each factor. We cannot resolve whether disruption of the CCN connectivity following stroke causes depressive features, or whether depressive features result in the decrease in connectivity within the network. Future studies should investigate causal relationships between depression and connectivity.

In addition, depression correlates of post-stroke depression have been shown to change over time^11,50^. Here we present resting-state correlates of depressive features at only one time point, 3 months post stroke. However, this time point represents an early period post stroke. Resting-state markers in later post-stroke stages should be further explored to understand the disorder trajectory relative to the CCN state.

### Summary

We examined the resting-state connectivity of the CCN in a stroke population with depressive features, compared to those without. We show that lower connectivity between the left DLPFC and the right SMG is associated with post-stroke depressive features. While the left DLPFC has been previously associated with cognitive impairment in depression in several lesion-symptom mapping studies, we now show that post-stroke depressive features are associated with a network connectivity problem, without necessarily involving a stroke lesion in DLPFC. We provide new clues about what represents a CCN resting connectivity marker in stroke participants with and without depressive features.
